# Immediate and long-term microshear bond strength of resin-based cements to core build-up materials

**DOI:** 10.4317/jced.58515

**Published:** 2021-10-01

**Authors:** Fernanda C. Lacerda, Waldemir F. Vieira-Junior, Paulo E. de Lacerda, Cecília P. Turssi, Roberta T. Basting, Flávia L. do Amaral, Fabiana MG. França

**Affiliations:** 1 DDS, MSc, PhD. Faculdade São Leopoldo Mandic, Instituto de Pesquisas São Leopoldo Mandic, Campinas/SP, Brazil

## Abstract

**Background:**

To evaluate the microshear bond strength (μ-SBS) between resin-based cements and core build-up materials after water storage.

**Material and Methods:**

Cylinders (1x1 mm) of conventional dual-cure resin cement (RelyX ARC, 3M ESPE), universal dual-cure resin cement (RelyX Ultimate, 3M ESPE) or self-adhesive resin cement (RelyX U200, 3M ESPE) were adhered to disks (6x2 mm) made with commercial core build-up materials (Bis-Core, Bisco or LuxaCore Z, DMG) or conventional nanocomposite resin (Filtek Z350 XT, 3M ESPE). The specimens (n=10) were submitted to μ-SBS test using a universal testing machine and fracture pattern analysis at 48 hours or after 9 months of water storage. The data were evaluated by three-way ANOVA for repeated measures and Tukey’s test (α=0.05).

**Results:**

After 48 hours, regardless of the resin-based cement used, the μ-SBS to the conventional resin composite (Filtek Z350 XT) was greater compared to other materials. However, after 9 months, there was a statistically significant decrease in μ-SBS values between the conventional resin composite and evaluated luting agents. The μ-SBS values of core build-up commercial materials (Bis-Core and LuxaCore Z) did not change over time. Regardless of the composite used and storage time, there was no statistically significant difference between the resin-based cements. After 48 h, the most prevalent failure was mixed for all groups. However, after 9 months, the core build-up materials showed a predominantly mixed fracture pattern whereas the resin composite presented an adhesive pattern.

**Conclusions:**

Commercial core build-up materials achieved stable bonding performance with resin-based cements over time.

** Key words:**Composite resins, resin cement, dental restoration failure.

## Introduction

Teeth extensively destroyed with a lost coronal structure can be used to support a restoration or other rehabilitations in order to restore function, aesthetics and comfort to the patient (1-4) when appropriate techniques, management and treatments are applied. Thus, post-and-core restorations are necessary depending on the degree of dental destruction, tooth affected, occlusion, root canal configuration and other clinical variables (3,5-7).

The growing demand for aesthetic restorations and minimally invasive treatments has led to the development of materials/products such as glass fiber posts that have an elasticity module similar to dentin, which reduces the risk of dental fracture (8-10). The glass fiber posts enable homogeneous root reconstruction allowing the absorption of occlusal loads through adhesive luting agents (11), which adheres to enamel and dentin (12). The core build-up restores the volume of the coronary portion and supports subsequent rehabilitation through indirect restoration (7,10,13).

Commonly, resin composites are materials used to performed core build-up due to their mechanical resistance, ease of use and adhesion to the dental structure (14-16). To optimize the clinical time, commercial core build-up materials (dual-cured resin-based composites) were developed to simultaneously cement the post and make the core build-up. This unique clinical step enables the formation of a monoblock reducing the technical sensitivity and consequently could decreases failures in adhesion between the resin cement and the material used for performing the post-and-core restorations (17-19).

Adhesive cementation of ceramic crowns increases the fracture resistance of the core-crown complex (20) and directly correlates with long-term clinical success. Previous studies have evaluated the adhesive relationship between post and luting agent (9,18), post and core material (21), luting agent and enamel/dentin (12), tooth and core materials (22), and crown and core materials (23). However, there is a lack of investigations about the bonding relationship between cement and the material used to perform the core build-up restorations.

Resin-based cements can be categorized as conventional, which require the use of adhesive systems to promote adhesion to dental structures (24,25); self-adhesive cements, which are hybrid materials that adhere to dental structures and restorative materials without requiring the application of adhesives or dental substrate pretreatment (25); and universal cements designed for use in different conditioning techniques, either by total-etch, self-etch or selective-enamel-etch modes. Furthermore, the camphorquinone-based photo-initiator system in some cement has been replaced by amine-free materials to preserve the color stability of these materials. Camphorquinone requires a co-initiator, typically amines, which interact and release free radicals responsible for initiating the polymerization reaction. Specifically, tertiary amines are highly reactive molecules and oxidize over time, producing a yellowish effect or lower color stability in resin-based cements (26,27).

Considering the different cements available on the world market, comparative studies of the bond strength between resin-based cements and core build-up materials are necessary. Therefore, the objective of the present study was to evaluate the bond strength between dual resin cements (conventional, amine-free/universal and self-adhesive) and commercial core build-up materials or conventional nanocomposite resin. The null hypotheses tested were as follows: 1- The build-up materials and resin composite would not differently influence the microshear bond strength values; 2- the storage time would not influence the microshear bond strength values between the different resin cements and core build-up materials evaluated; 3- the resin-based cements studied would not influence the values of bond strength to the core build-up materials.

## Material and Methods

-Study design

The experimental units for this *in vitro* study were cylinders/pillars obtained from resin cements that were adhered to disks of commercial core build-up materials or conventional nanocomposite resin. The variables studied were the bond strength values verified by the microshear test (μ-SBS, MPa) and the fracture pattern assessed qualitatively (%). The following factors were studied.

(I) Core build-up materials: commercial core build-up materials (Bis-Core, Bisco or LuxaCore Z, DMG) and conventional nanocomposite resin (Filtek Z350 XT, 3M ESPE)

(II) Resin cements: conventional dual-cure resin cement (RelyX ARC, 3M ESPE), amine-free dual-cure resin cement (RelyX Ultimate, 3M ESPE) or self-adhesive resin cement (RelyX U200, 3M ESPE) 

(III) Storage time: 48 hours and 9 months.

The sample calculation was conducted (G* Power 3.1.5, Heine, Universität Dusseldorf, Germany) using the analysis of variance (ANOVA) model. For the effect size of 0.15, obtained from the data collected in a pilot study (n = 4), significance level of 5% and power of 80%, the sample calculation indicated the need for a total of 10 specimens per group. Thus, the samples were randomly divided into nine groups (n = 10) according to the factors under study and are specified in Table 1. The composition and application method of the materials are presented in Table 2.

**Table 1:**
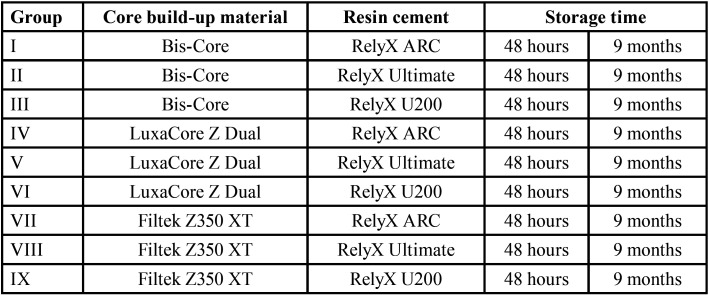
Group division (n = 10) considering the study factors: core build-up materials, resin cements, and storage time.

**Table 2:**
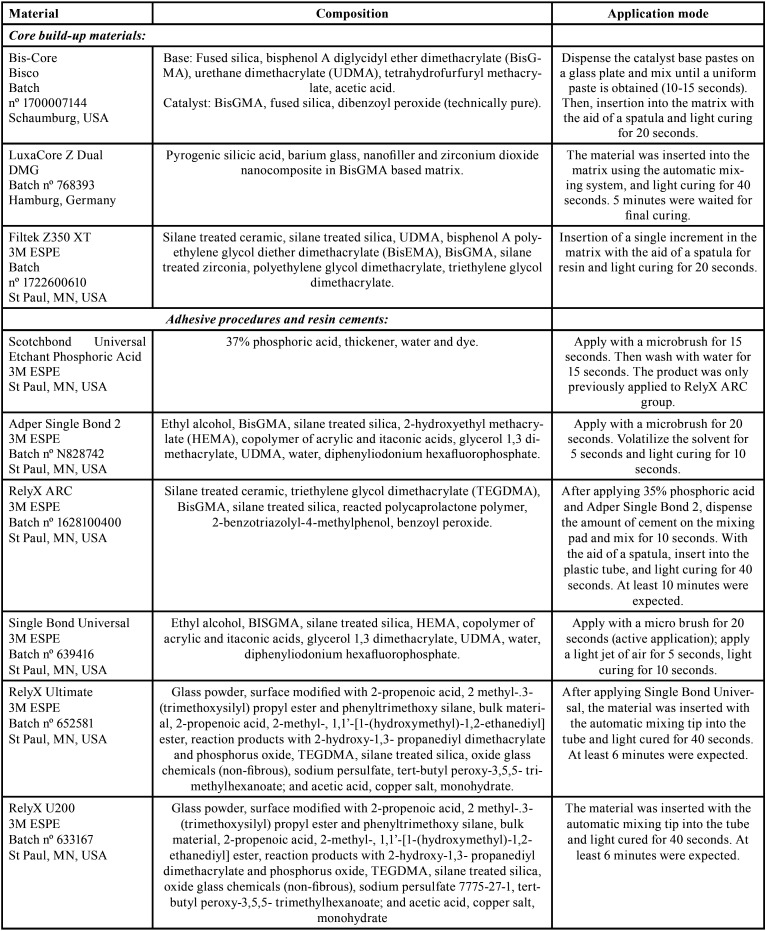
Description, composition and application mode of materials used in present study.

Specimen preparation: disks of core build-up materials 

Under a glass cover slip and a polyester strip, a matrix 6 mm in diameter and 2 mm in height was positioned. The materials were inserted into the matrix as follows: LuxaCore Z with the aid of a mixing tip, Bis-Core and resin composite (Filtek Z350 XT) were applied in a single layer with the aid of a spatula. Then, another polyester strip and another cover slip were placed, and the material was photoactivated with LED (Valo, Ultradent) for 20 seconds (Bis-Core and Filtek Z350) or 40 seconds (LuxaCore Z Dual) with irradiation of 1400 mW/cm2.

After this step, the disks were embedded in epoxy resin using PVC cylinders and their surface was smoothed and polished in a polishing machine (Politriz, Arotec Ind. E Comércio, São Paulo, Brazil) with 600-grit sandpaper. The adhesive process was carried out after specimens were made according to the orientation of the manufacturer or type of cement evaluated in the current study model. Thus, when RelyX ARC (3M ESPE) was used, the adhesive applied was Adper Single Bond 2 (3M ESPE); when RelyX Ultimate was used, the adhesive system applied was Single Bond Universal (3M ESPE); and when cement RelyX U200 (3M ESPE) was used, which is self-adhesive no adhesive process was performed. Table 2 describes the application steps for each material, including the adhesive systems.

Specimen preparation: cylinders/pillars of resin cements

After preparing the disks, two plastic tubes (1 x 1 mm; diameter x height) were fixed on the surface of the specimen. The dual-cure resin cements were inserted into the tubes with the aid of an insertion spatula (RelyX ARC) or automatic mixing tip (RelyX Ultimate and RelyX U200). After excesses were removed, photoactivation was carried out for 20 seconds (Valo, Ultradent). After 1 hour at room temperature, the tubes were gently removed using a scalpel blade (No. 15, Med Goldman Indústria e Comércio Ltda., Santa Catarina, Brazil). In each specimen, two cylinders of the resin cement were obtained, one for the microshear test after 48 hours and the other after 9 months of storage in distilled water at 37ºC, which was renewed every week.

-Microshear strength test and fracture pattern analysis

**Figure 1:**
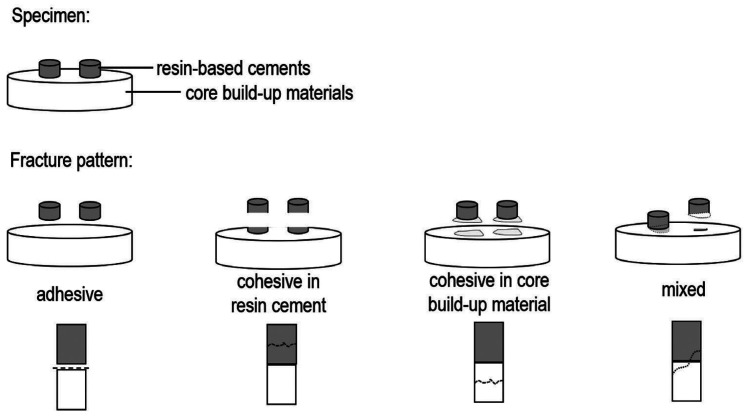
Representative scheme of the fracture patterns considered in the present study.

The microshear strength test of specimens was carried out in a universal machine (EZ test, model DL-2000, Shimadzu) with a load of 50 Kgf at 0.5 mm/min, until the resin cement cylinders ruptured. For this, a stainless-steel wire was used that surrounded the cylinder. The microshear strength values were recorded in Newton (N) and transformed into megapascal (MPa). After the rupture of the resin cement cylinders, the surfaces of the disks were observed at 30x magnification using a stereoscope (EK3ST, Eikonal, São Paulo, Brazil) to determine the fracture pattern, classified as adhesive/interface, cohesive in resin cement, cohesive in core build-up material, or mixed (Fig. 1).

-Statistical analysis

The microshear values (MPa) were submitted to three-way repeated measures ANOVA to investigate the effect of core build-up material, resin cement, storage time, and the triple or double interactions between the factors under study. The Tukey’s test was used to evaluate the multiple comparisons. The fracture pattern results (%) were qualitatively described. The analyses were conducted using the SPSS 23 program (SPSS Inc., Chicago, IL, USA), adopting a significance level of 5%.

## Results

According to the results presented in Table 3, there was no statistically significant triple interaction between the factors under study (core build-up materials, resin cement and storage time; *p* = 0.238). Nevertheless, considering the double interactions, the factors of core build-up materials and storage time were statistically significant (*p* = 0.023). After 48 hours, the conventional nanocomposite resin (Filtek Z350 XT) obtained bond strength values significantly higher than those found in commercial core build-up materials (Bis-Core and LuxaCore Z), which did not differ from each other.

**Table 3:**
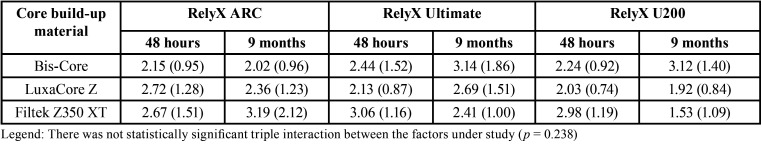
Means (SD) of microshear bond strength values (MPa) according to core build-up materials, resin cements, and storage time.

On the other hand, after 9 months, the bond strength of the Bis-Core group was significantly higher than that of the conventional nanocomposite resin (Table 4). After 9 months of water storage, LuxaCore Z presented intermediate values, which were not significantly different from those of other composites. Considering the effect of storage time for each core build-up material, the storage time caused a statistically significant reduction in bond strength values only for the conventional resin composite (Filtek Z350 XT). For Bis-Core and LuxaCore Z materials, the bond strength values did not alter over time.

**Table 4:**
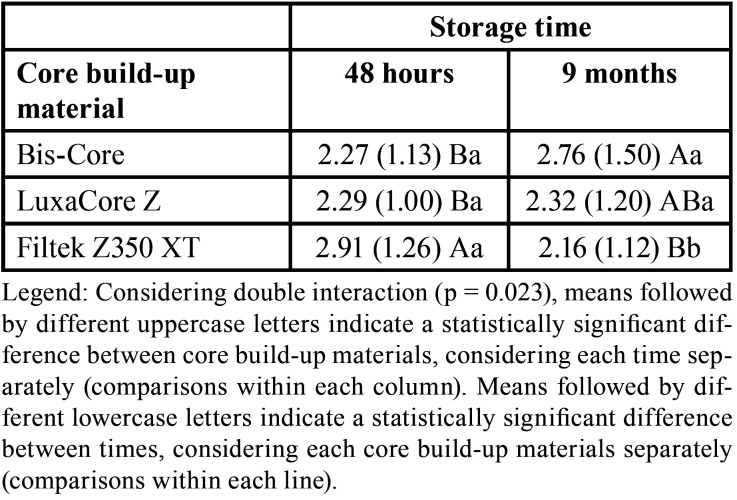
Means (SD) of microshear bond strength values (MPa) according to core build-up materials and storage time regardless of resin cement used.

**Figure 2:**
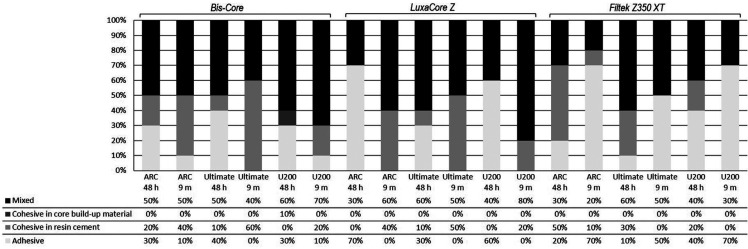
Fracture pattern (%) between core build-up material and resin cements after 48 hours and 9 months of water storage.

Regarding the fracture pattern (Fig. 2), after 48 hours in storage, the fracture pattern between the core build-up materials and resin-based cements was predominantly mixed. However, after 9 months of storage, the commercial core build-up materials (Bis-Core and LuxaCore Z) showed a predominantly mixed failure, while the conventional resin composite was adhesive/interface. After 9 months, no pattern of cohesive fracture in core build-up materials was observed.

## Discussion

The core build-up materials have dual functionality, being inserted in a single layer to cement the post, and reconstruct the crown in the same clinical act (7,18). This technique reduces the time required to perform the procedure, the number of materials used, the technical sensitivity and incompatibilities between luting agent and core build-up material (11). In the present study, the influence of core build-up material on the microshear bond strength of resin cements was evaluated over time. The materials evaluated were LuxaCore Z, Bis-Core and a nanocomposite resin (Filtek Z350 XT), which was considered a control group for having adequate biomechanical behavior and resistance, ease of handling and adhesion to the dental structure (14,15).

After 48 hours, the nanocomposite resin (Filtek Z350 XT) showed higher bond strength values than the core build-up materials, followed by Bis-Core and LuxaCore Z, rejecting the first null hypothesis. This probably happened due to the interaction between the organic polymeric matrix and the inorganic filler present in the composition of each material studied. The polymerization reaction of Filtek Z350 XT is triggered with the absorption of blue light by camphorquinone and the reaction of free radicals generated with the double bonds of dimethacrylates (Bis-GMA, UDMA and TEGDMA, Table 1) present in the organic polymeric matrix. The network formed is highly reticulated, demonstrating resistance values of compressive strength and flexural strength equivalent to or greater than those of other conventional composites (14,17).

Considering the commercial core build-up materials, LuxaCore Z is composed of nanofiller and zirconium dioxide nanocomposite in a Bis-GMA-based matrix (filler content = 50%), whereas Bis-Core is composed by fused silica (10-30%), Bis-GMA (5-10%), UDMA (5-10%), tetrahydrofurfuryl methacrylate (1-5%) and acetic acid (< 1%). These materials depend on a dual polymerization reaction occurring due to light curing associated with the presence of an initiator-accelerator system. The dimethacrylate molecules present in the organic polymeric matrix, such as Bis-GMA and UDMA, have double carbon bonds at their ends, and after absorption of optical radiation, the photoinitiator (camphorquinone) absorbs blue light and releases free radicals, polymerizing and reticulating the system to provide a hardening of the resin-based materials in the external areas. Then, the self-curing mechanism starts with the aromatic or aliphatic amines present in the catalyst paste that reacts with the peroxide of the universal paste to produce free radicals, which complement the action at the unreacted carbon double bond sites, ensuring the polymerization of the material in the deepest areas that receive insufficient light intensity (14,17,28).

Commonly, the commercial core build-up materials have a lower percentage of filler content, which could cause a decrease in mechanical properties compared to conventional resin composite, even with dual polymerization. This difference in inorganic composition explains the lower bond strength between the tested resin cements adhered to the conventional nanocomposite and other materials evaluated after 48 hours. Karakis *et al*. (17) suggest that the self-curing mechanism for double-activated resin-based materials is slower and less effective in terms of monomer conversion than light activation. Areas with different concentrations of carbon double bonds can coexist in the same polymer and affect the amount of crosslink density, leading to heterogeneity in the polymeric network and a reduction in the overall crosslink density, corroborating the result found after 48 hours.

Nevertheless, after 9 months, the Bis-Core material showed higher bond strength values compared to conventional resin composite, rejecting the second null hypothesis. The maintenance of the bond strength values of the commercial core build-up materials over time can be attributed to the dual cure that occurs in these materials (7), which is not found in Filtek Z350, which presented loss of bond strength over time (18). The stable bonding performance of Bis-Core and LuxaCore Z could be due to the concentration of dimethacrylate monomers (14), justifying the prevalence of mixed fracture patterns in the samples, as shown in Figure 2. Comparing the two commercial core build-up materials, LuxaCore Z showed intermediate bond strength values compared to Bis-Core, probably due to zirconium dioxide used as filler, which can reduce light transmission during photoactivation. Thus, reductions in the monomer conversion consequently decrease the mechanical properties of the material, leading to a less stable bond. Moreover, the silanization process of zirconium dioxide particles of LuxaCore Z could not be as efficient as Bis-Core, which contains silica (18).

On the other hand, after 9 months, the decrease in the bond strength values of the resin composite groups might have been due to water sorption, polymer network and filler content. Water sorption by polymers is a diffusion-controlled process, in which the water absorbed by the polymeric matrix can cause the breakdown or softening of bonds between the polymeric matrix and inorganic fillers, characterizing a hydrolytic degradation (29). The resin composite has higher filler content, and unreacted monomers or filler particles leached when there was water sorption by the polymeric matrix and hydrolytic degradation can occurs, resulting in weight and volume loss (24). The Filtek Z350 resin composite and the resin cements used in the study have a similar amount of silane-treated silica (1-10%), which can promote a low crosslink density beneficial to hydrolytic degradation (17), justifying the decrease in bond strength values after storage and the predominance of the adhesive fracture pattern (Fig. 2). However, the results of fracture pattern are influenced not only by the characteristic of adhesive interface but also by other mechanical properties of the studied materials, especially when the failures were categorized as cohesive in the material.

The present study design considered evaluating dual resin cements of different compositions that are available on the world market and used with different tooth preparations. The bond strength values found in the present *in vitro* study demonstrated that Bis-Core and LuxaCore materials can be used with different resin cements (conventional or amina-free dual-cure and self-adhesive resin cement), accepting the third null hypothesis. Although the results of the present study are interesting, the success of the restorative treatment is not exclusively based on the bonding performance of core material to the resin cement used for luting the indirect restoration; other material properties and clinical factors such as dental structure, selective wear of the dental substrates, restorative material to support occlusal forces, periodontal health, and core design are also important (30). Thus, further studies and clinical trials evaluating these materials are encouraged, especially because it is not possible to infer that this small numerical difference between groups for Mpa values seems to be considered clinically relevant.

## Conclusions

Despite higher initial values of microshear bond strength for conventional resin composite, commercial core build-up materials (Bis-Core and LuxaCore Z) presented more sTable bond strength values to different resin-based cements after 9 months of water storage.
